# Peptidomic characterization and bioactivity of Protoiurus kraepelini (Scorpiones: Iuridae) venom

**DOI:** 10.3906/biy-1804-35

**Published:** 2018-12-10

**Authors:** Tuğba SOMAY DOĞAN, Naşit İĞCİ, Ayşenur BİBER, Selin GEREKÇİ, Hepşen Hazal HÜSNÜGİL, Afife İZBIRAK, Can ÖZEN

**Affiliations:** 1 Center of Excellence in Biomaterials and Tissue Engineering, Middle East Technical University , Ankara , Turkey; 2 Central Laboratory, Middle East Technical University , Ankara , Turkey; 3 Department of Biology, Faculty of Science, Hacettepe University , Ankara , Turkey; 4 Department of Molecular Biology and Genetics, Faculty of Sciences and Arts, Nevşehir Hacı Bektaş Veli University , Nevşehir , Turkey; 5 Graduate Program of Biotechnology, Middle East Technical University , Ankara , Turkey; 6 Science and Technology Research and Application Center, Nevşehir Hacı Bektaş Veli University , Nevşehir , Turkey

**Keywords:** Scorpion venom, peptide, peptidomics, antimicrobial effect, cytotoxicity, mass spectrometry

## Abstract

Protoiurus kraepelini is a scorpion species found in parts of Turkey and Greece. In this study, the peptide profile of its venom was determined for the first time. The electrophoretic profile of the crude venom showed a protein distribution from 2 to 130 kDa. MALDI-TOF MS analysis of the venom peptide fraction yielded 27 peptides between 1059 and 4623 Da in mass. Several ion channelblocking and antimicrobial peptides were identified by peptide mass fingerprinting analysis. Cytotoxic and antimicrobial effects of the venom were also demonstrated on Jurkat cells and Escherichia coli, respectively. As the first peptidomic characterization study on P. kraepelini venom, this report lays the foundation for detailed future studies that may lead to the discovery of novel bioactive peptides.

## 1. Introduction

A wide variety of species have been producing toxins over millions of years in order to capture prey or as a defense mechanism. Some of these active compounds have been used in the development of new drugs for the treatment of various diseases (Harvey, 1995; Clardy and Walsh, 2004; Newman and Cragg, 2007) . Such pharmacologically active biomolecules show their biological activity by inducing or inhibiting apoptosis and angiogenesis, inhibiting protein synthesis, or displaying antimicrobial effects. Among the animals that produce pharmacologically active molecules capable of interfering with human cellular physiology, special attention has been given to venomous reptiles and invertebrates such as scorpions, bees, wasps, spiders, ants, caterpillars, and sea snails (Lewis and Garcia, 2003; Heinen and da Veiga, 2011). Animal venoms are rich sources of bioactive molecules that have evolved to express high affinity and selectivity for various biological targets, such as ion channels, receptors, coagulation factors, and transporters (Lewis and Garcia, 2003; Tedford et al., 2004; Fry et al., 2009) . Venoms are composed mostly of proteins and peptides, encompassing a large variety of structures and modes of action. In particular, the afinity and specificity of venom peptides, their feasibility for chemical synthesis and/or recombinant production, and their resistance to proteolytic degradation (especially disulfide-rich peptides) are attributes that have made them attractive drug candidates (Lewis and Garcia, 2003; Olivera, 2006; Newman and Cragg, 2007).

Scorpion venom is a mixture of approximately 70–600 different compounds such as polypeptides, nucleotides, lipids, biogenic amines, heterocyclic compounds, and inorganic salts (Possani et al., 2000; Quintero-Hernández et al., 2013; Ortiz et al., 2015). Although there are over 1700 species of scorpions, only a few dozen have been well characterized. As of 12 January 2018, 772 scorpion venom toxins (963 proteins/peptides in total) were described in the UniProt Animal Toxin Annotation Project database (http://www.uniprot.org/program/Toxins), which corresponds to less than 1% of the estimated total number. Various scorpion venom peptides have been shown to be a valuable source for drug discovery due to their ion channel-blocking, anticancer, and antimicrobial activities (Heinen and da Veiga, 2011; Ortiz et al., 2015).

Turkey has a rich scorpion fauna with many endemic species. Studies on the venomic characterization of scorpions found in Turkey have focused on Androctonus crassicauda, Buthacus macrocentrus, and Mesobuthus gibbosus, and several toxins have been characterized (Caliskan et al., 2006, 2012; Diego-García et al., 2013) . Recently, peptide diversity and cytotoxic and antimicrobial effects of Leiurus abdullahbayrami were also investigated by our group (Erdeş et al., 2014).

Protoiurus kraepelini (family Iuridae) is a scorpion species mainly distributed in Antalya, Isparta, Konya, Karaman, Mersin, and Muğla provinces of Turkey and Megisti Island of Greece (Soleglad et al., 2012; Yağmur et al., 2016) . There has been no detailed biochemical study on its venom previously. The aim of this study was to characterize P. kraepelini venom, focusing on its peptidomic content and bioactivity.

## 2. Materials and methods

### 2.1. Specimen collection

**Figure 1:**
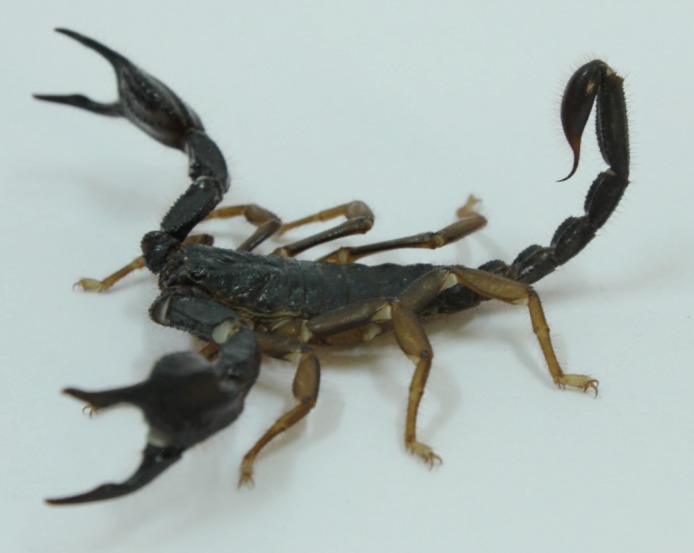
Adult Protoiurus kraepelini in captivity. Photograph by the second author.

Scorpions were collected in Alanya, Turkey (Figure 1). They were maintained in plastic boxes and fed mealworms.

### 2.2. Venom milking

Venom was milked from adult individuals by electrical stimulation (15 V) applied to the telson. Venom samples were collected and pooled in polypropylene tubes, diluted with double distilled water, and centrifuged at 15.000 × g for 15 min at 4 °C. The supernatant was then transferred to a new tube, lyophilized by freeze-drying, and stored at –80 °C.

### 2.3. Protein content determination

Protein contents of the crude venom and fractions were determined using the Bio-Rad Quick Start Bradford Protein Assay Kit according to the instructions of the manufacturer.

### 2.4. Electrophoresis

Tris-glycine sodium dodecyl sulfate-polyacrylamide gel electrophoresis (SDS-PAGE) was performed using 4% stacking and 10% resolving gels in a Tris-glycine buffer (pH 8.3) containing 0.01% SDS at a constant current (30 mA). The Tris-Tricine SDS-PAGE method is used for the separation of low-molecular-mass proteins according to the procedure of Schägger (2006).

### 2.5. Chromatographic separation

A Varian Prostar HPLC equipped with an autosampler and diode array detector was used for the fractionation of the venom components.

#### 2.5.1. Size exclusion chromatography (SEC)

Fifty microliters of 50 mg/mL crude venom was injected into a SEC column (Tosoh Bioscience TSK G2000SW, 5 mm × 600 mm, 12.5 nm pore size) and run for 60 min with a 0.5 mL/min flow rate. Peptide and protein fractions were collected, freeze-dried, and stored at –80 °C for further analyses.

#### 2.5.2. Reversed-phase chromatography (RPC)

The venom peptide fraction from SEC chromatography was diluted to 30 µg/µL with solution A (0.1% triflouroacetic acid [TFA] in deionized water) and injected (50 µL) into a C18 reversed-phase column (Vydac 218TP54, 4.6 mm × 250 mm, 300 Å pore size) followed by a run for 90 min with 0.7 mL/min flow rate. A linear gradient of solution A to 60% solution B (0.1% TFA in acetonitrile) was used for the elusion. Collected fractions were freeze-dried and stored at –80 °C for further analyses.

### 2.6. Mass determination

#### 2.6.1. Electrospray ionization (ESI) mass spectrometry

An Agilent 6530 liquid chromatography-electrospray ionization-time of flight mass spectrometry (LC-ESI-TOF MS) system connected to an Agilent 1200 HPLC was used for the determination of the molecular weight of venom peptides. The SEC peptide fraction (50 µL, 30 µg/mL) was injected into a C18 reversed-phase column (Agilent ZORBAX Eclipse XDB-C18, 4.6 mm × 150 mm, 5 µm) and run with identical settings as described in Section 2.5.2. Eluted fractions were sent to the ESI MS system. TOF parameters were set to 2000 V with positive-ion mode and capillary voltage was set to 5000 V. Data interpretation was conducted using Agilent Mass Hunter Workstation Qualitative Analysis software.

#### 2.6.2. Matrix-assisted laser desorption ionization (MALDI) mass spectrometry

The lyophilized peptide fraction was dissolved in 50% acetonitrile-0.1% TFA, and 0.5 µL of this sample solution was mixed with an equal volume of α-cyano4-hydroxycinnamic acid (CHCA, Sigma-Aldrich) or sinapic acid dissolved in 60% acetonitrile-0.3% TFA and then spotted onto the MALDI target plate by dry-droplet method. Mass spectra was acquired on a MALDI-TOF mass spectrometer (Waters, Eschborn, Germany) operated in reflectron positive-ion mode after an external calibration using bovine insulin oxidized B chain (monoisotopic mass = 3494.6513 Da) and angiotensin 1 (monoisotopic mass = 1286.6853 Da). Data interpretation was performed using MassLynx 4.0 software (Waters).

#### 2.6.3. Peptide mass fingerprinting (PMF)

The SEC peptide fraction was separated by PAGE and stained with colloidal Coomassie Blue (Bio-Rad), and then two major bands were excised and sliced into small pieces by sterile scalpel and subjected to in-gel digestion as previously described (Igci and Demiralp, 2012). Tryptic peptides in each band were measured using a highresolution nano-LC quadrupole TOF-MS/MS system (Synapt G2, Waters, Eschborn, Germany) operated in positive-ion and V analyzer mode. Capillary voltage was 3 kV. A survey TOF scan was recorded for the mass range of 50–2000 Da. Spectra were manually interpreted and peak lists were generated. The MS-Fit Engine on the Protein Prospector platform (http://prospector.ucsf. edu/prospector/mshome.htm) and the Mascot Search Engine (http://www.matrixscience.com/) were used for PMF analysis and all searches were performed against the UniProtKB database. Carbamidomethylation of cysteine was chosen as a modification, and maximum missed cleavage and peptide tolerance were set to 1 Da in all searches. Taxonomy was selected as “Scorpions” in MS-Fit and “Other Metazoa” in Mascot searches.

### 2.7. Bioactivity screening

An Alamar Blue (Molecular Probes, Invitrogen) assay was employed for cytotoxicity measurements. Growth inhibition measurements were based on the broth dilution method (Wiegand et al., 2008; Vassilevski et al., 2010) and included Staphylococcus aureus (ATCC 6538), Escherichia coli (ATCC 25922), and Candida albicans (DSMZ 1386) strains.

## 3. Results

### 3.1. Protein content and electrophoretic profile of the venom

**Figure 2:**
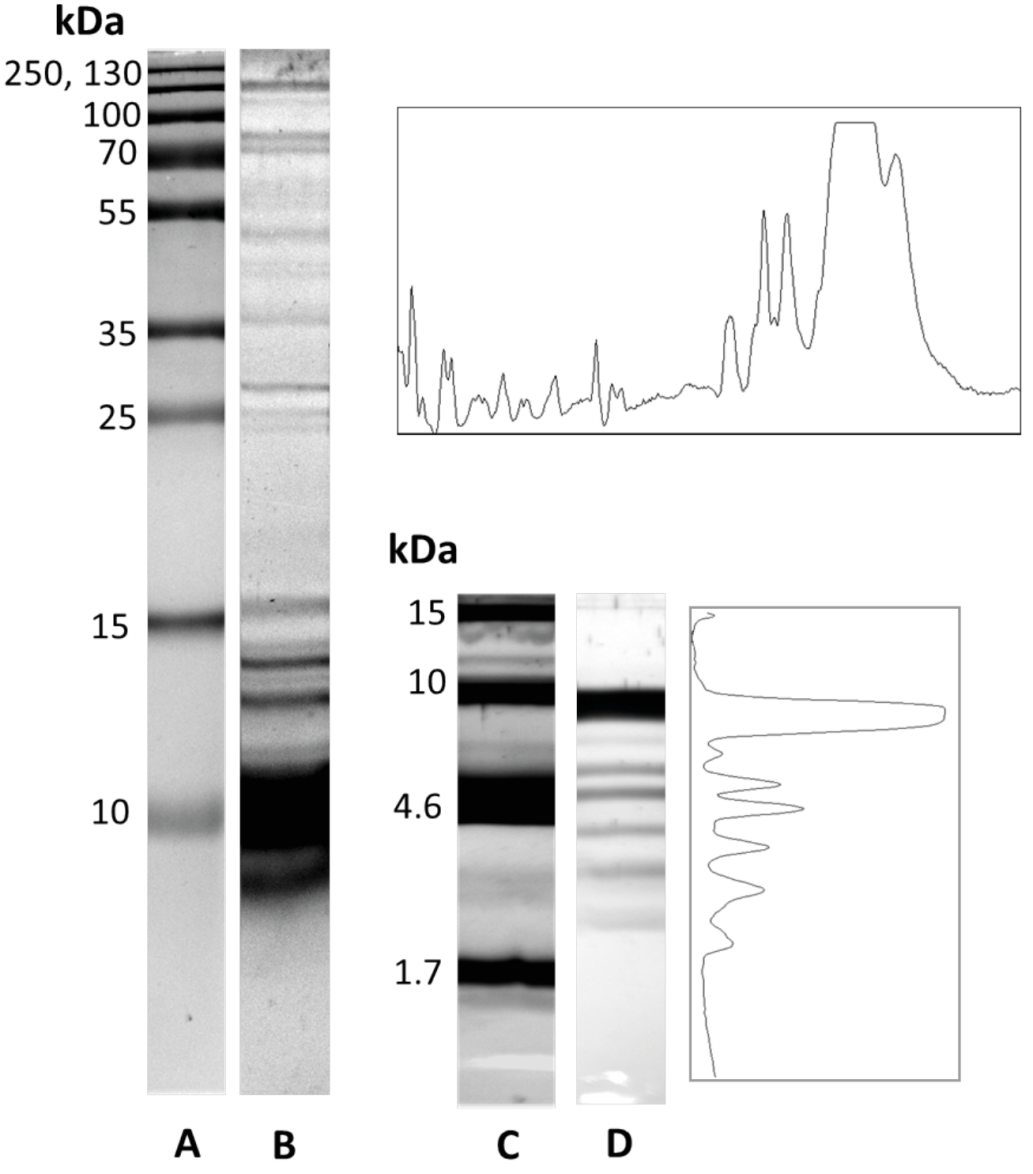
Electrophoretic separation of the crude venom (B) and peptide fraction (D) with accompanying densitometric curves. (A) and (C) refer to molecular weight standards.

The amount of protein based on dry weight of the crude venom was found to be 70% (w/w) by Bradford protein assay. Venomic protein/peptide bands from ~6 kDa up to ~150 kDa were observed on the Tris-glycine gel (22 bands in total), with a major band at ~10 kDa (Figure 2). The peptide fraction of the venom yielded 7 observable bands under 10 kDa on Tris-Tricine SDS-PAGE.

### 3.2. Venom fractions

**Figure 3:**
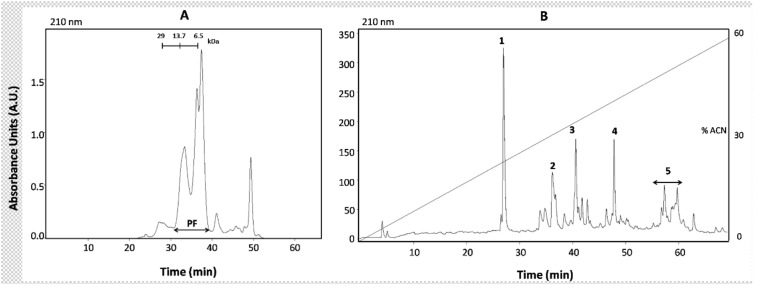
HPLC fractionation of P. kraepelini venom: A) Size exclusion chromatogram indicating the peptide fraction (PF); B) reversed-phase chromatogram of the peptide fraction. Peak sets tested in the bioactivity assays are numbered on the plot.

As shown in Figure 3, three major peptide peaks between 2.5 and 30 kDa were collected, pooled, and labeled as the venom peptide fraction (PF) after multiple SEC runs. The peptide fraction was further fractionated by RP-HPLC and five peak sets were pooled separately for bioactivity assays.

### 3.3. Mass profile of the peptide fraction

**Figure 4:**
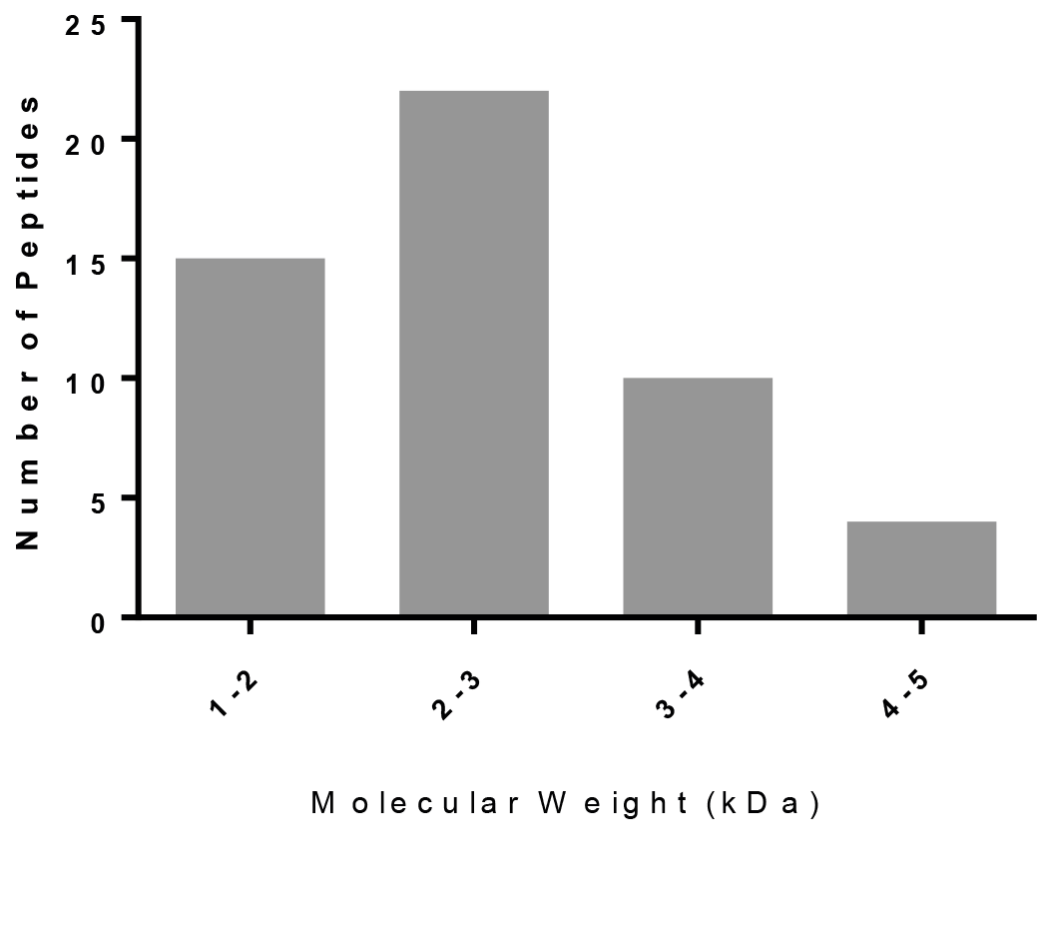
Molecular weight distribution of the short peptides detected by mass spectrometry measurements (LC-ESI-TOF and MALDITOF).

LC-ESI-TOF MS and MALDI-TOF MS were used in combination for the molecular weight determination of the proteins in the venom peptide fraction. The deconvoluted molecular weights of 25 peptides resulting from MALDITOF MS analysis and 27 peptides from LC-ESI-TOF MS are summarized in Supplementary Table S1. In total, 48 unique masses were found excluding the shared peptide masses (±1 Da) detected in both methods. The molecular weight distribution histogram of the detected peptides (1059 to 4623 Da) is provided in Figure 4.

### 3.4. Identified venom peptides

PMF analysis yielded the identity of 15 peptides/proteins in the P. kraepelini venom (Table 1). Among the peptides/ proteins identified by PMF analysis, the major peptide/ protein family was scorpion venom K+ ion channelblocking peptides (KTx). Nine peptides were identified as belonging to the alpha, beta, and gamma KTx families. Additionally, phospholipase A2 (PLA2), a fragment of sodium channel-modifying neurotoxin Cex6 [Q86QV1], and antimicrobial peptides opiscorpine-1 [Q5WR03] and opiscorpine-3 [Q5WQZ7] were identified as venomic components.

**Table 1:** Proteins identified in P. kraepelini venom by mass fingerprinting analysis.

Protein ID	Accession no	Theoretical Mw (kDa)	Score	Number of matched whole peptide masses	Sequence coverage (%)	Search engine	Species
Potassium channel toxin beta-Ktx 2	P86822	10.0	8.989	15	61	MS-Fit	Tityus serrulatus
Potassium channel toxin alpha-KTx 6.8	Q6XLL7	6.7	218	9	54	MS-Fit	Opistophthalmus carinatus
Potassium channel toxin alpha-KTx 6.8	Q6XLL7	7.1	31	7	45	Mascot	O. carinatus
Potassium channel toxin alpha-KTx 6.3	P59867	3.8	175	4	70	MS-Fit	Heterometrus spinifer
Potassium channel toxin alpha-KTx 2.6 (fragment)	P59849	3.6	169	4	52	MS-Fit	Centruroides limbatus
Potassium channel toxin alpha-KTx 4.4	P60210	3.8	133	5	48	MS-Fit	T. obscurus
Potassium channel toxin alpha-KTx 4.1	P0CB56	4.2	28	13	100	Mascot	T. stigmurus
Potassium channel toxin gamma-KTx 5.2	Q86QV1	5.5	24	8	68	Mascot	C. gracilis
Toxin KTx8	A9QLM3	7.3	22	6	26	Mascot	Lychas mucronatus
Potassium channel toxin alpha-KTx 6.7	Q6XLL8	7.2	21	6	32	Mascot	O. carinatus
Neurotoxin Cex6 (fragment)	Q68PG9	7.7	147	6	37	MS-Fit	C. exilicauda
Venom protein 30.1	P0CJ18	17.0	29.879	18	49	MS-Fit	L. mucronatus
Phospholipase A2	Q6T178	18.5	26.633	18	43	MS-Fit	Mesobuthus tamulus
Phospholipase A2 heteromtoxin	P0DMI6	18.2	5.642	13	25	MS-Fit	H. laoticus
Opiscorpine-3	Q5WQZ7	10.3	5.908	13	58	MS-Fit	O. carinatus
Opiscorpine-3	Q5WQZ7	10.7	24	13	58	Mascot	O. carinatus
Opiscorpine-1	Q5WR03	10.8	22	12	53	Mascot	O. carinatus

### 3.5. Cytotoxicity of the venom peptide fraction

**Figure 5:**
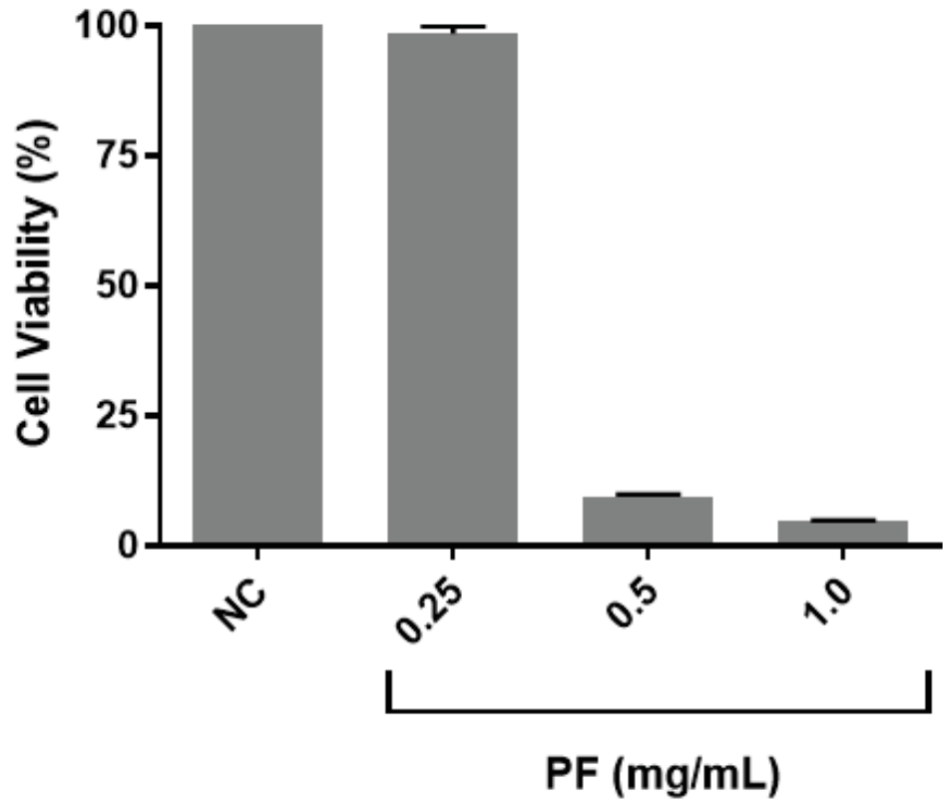
Cytotoxicity of the venom peptide fraction on Jurkat human T-cell leukemia cell line following 24 h of treatment. PF: Peptide fraction of the venom.

The peptide fraction of the venom showed dose-dependent cytotoxicity on Jurkat cells (Figure 5). While a significant decrease in cell viability at 0.5 and 1 mg/mL dose was observed, viability of the cells was not affected with 0.25 mg/mL treatment.

### 3.6. Antimicrobial activity

Crude venom (500 µg/mL) completely inhibited the growth of S. aureus and E. coli while no growth inhibition activity was observed for C. albicans even at 1 mg/mL treatment. Among the RP-HPLC fractions of the venom, only fraction 5 showed growth inhibition activity against E. coli at 300 µg/mL.

## 4. Discussion

In vivo toxic effects and lethality of Iurus dufoureius asiaticus venom were previously investigated using scorpion specimens collected in Muğla Province, Turkey (Ozkan et al., 2007). According to the latest taxonomy, this population is now named Protoiurus kraepelini (Soleglad et al., 2013; Yağmur et al., 2016) . Ozkan et al. (2007) identified eight major protein bands between 29 and 116 kDa in the venom without detailed information on the lowmolecular-weight protein content of the venom. Alpagut Keskin and Koç (2006) studied the venom proteins of I. d. asiaticus from Aydin Province, Turkey, using Tricine SDS-PAGE and observed 28 protein bands between 6.5 and 205 kDa, with the densest bands being below 15 kDa and at ~70 kDa. After the taxonomic revision of the iurids, the Aydin population of I. d. asiaticus was renamed Iurus kinzelbachi, which can be considered to be related to P. kraepelini (Soleglad et al., 2013; Yağmur et al., 2016) . In our study, Tris-glycine SDS-PAGE experiments resulted in 22 visible protein/peptide bands between ~10 and 150 kDa for crude P. kraepelini venom, with the most intense band at ~10 kDa. Since Tricine SDS-PAGE enables a higher separation of low-molecular-weight peptides (Schägger, 2006), we used this method with 16% gel and resolved the major 10 kDa glycine PAGE band into seven peptide bands between ~2 and 10 kDa. The major band appeared around 10 kDa, indicating the presence of short- and long-chain neurotoxins. Although Ozkan et al. (2007) did not report a major band below 15 kDa in P. kraepelini venom, our electrophoresis and SEC results clearly showed that most of the polypeptides in P. kraepelini venom have a molecular weight below 15 kDa, similar to many other scorpion venoms (Alpagut Keskin and Koç, 2006; Rodríguez de la Vega et al., 2010; Erdeş et al., 2014) .

The combined mass spectrometry approach granted more detailed insight into the peptidomic content of venom. In the present study, 48 different masses were detected between 1059 and 4623 Da in the peptide fraction. The majority of the peptides were in the 1–2 or 2–3 kDa range, indicating the abundance of non-disulfide-bridged proteins (NDBPs). Recent mass fingerprinting studies have shown that many scorpion venoms contain lowmolecular-weight peptides as a major fraction (Rodríguez de la Vega et al., 2010; Ortiz et al., 2015). These lowmolecular-weight peptides (e.g., NDBPs) are well known for their antimicrobial activity (Almaaytah and Albalas, 2014; Harrison et al., 2014; Ortiz et al., 2015) . The peptides between 3 and 5 kDa in size are possibly disulfide-bridged short-chain neurotoxins, including Cl– and K+ ion channel blockers (Possani et al., 2000).

PMF analysis enabled us to identify K+ ion channel blocking KTx peptides (all alpha, beta, and gamma types) using both MS-Fit and Mascot search instruments. We also identified neurotoxin cex6 (fragment) [Q68PG9], which is classified as a sodium channel modifier. Another protein family that we identified with a high score is phospholipase A2 (PLA2). PLA2 has been identified in a limited number of scorpion venoms and it is also found in snake and bee venoms (Igci and Demiralp, 2012; Incamnoi et al., 2013). PLA2 enzymes show diverse pharmacological and biological activities. Moreover, we identified opiscorpine, a small cationic antimicrobial peptide related to defensins (Zhu and Tytgat, 2004) . Tandem mass spectrometry based approaches yield better identifications, especially for nonmodel organisms (e.g., scorpions) with limited sequence data (Bringans et al., 2008) . Further proteomic studies based on MS/MS fragmentation data could provide more detailed information about P. kraepelini venom.

Scorpion venoms are a rich source of antimicrobial peptides (Harrison et al., 2014; Ortiz et al., 2015). For instance, hadrurin is a cationic antimicrobial peptide purified from the venom of the Mexican scorpion Hadrurus aztecus (Torres-Larios et al., 2000) . This scorpion was in the family Iuridae, but it has now been renamed as Hofmannihadrurus aztecus and included in the family Caraboctonidae, a family related to Iuridae, within the superfamily Iuroidea (Fet and Soleglad, 2008).

Different antimicrobial peptides have been purified and characterized from the venom of many other scorpion species (Harrison et al., 2014). Antimicrobial peptides were also identified in P. kraepelini venom by PMF analysis in the present study. Thus, we investigated the antimicrobial activity of P. kraepelini crude venom and fractions via the hit discovery approach to confirm our mass spectrometrybased results. We detected antibacterial activity of crude venom (0.5 mg/mL) and reversed-phase peptide fraction 5 (RPF5) (0.3 mg/mL) against E. coli. Crude venom (1 mg/ mL) also inhibited the growth of S. aureus. We did not, however, observe an antifungal effect against C. albicans.

Although the antibacterial activity of P. kraepelini was not strong, these results confirmed our findings obtained from mass spectrometry-based analyses. The need for new antibiotics has become an urgent requirement in the world once again, because of the resistance developed by microorganisms (Harrison et al., 2014). Scorpion venoms present a rich molecular repertoire with antimicrobial properties. New prototypes of antimicrobial agents could be purified and characterized through further research on P. kraepelini venom.

We also assessed the cytotoxic potential of venom against a human T-cell leukemia cell line (Jurkat). The peptide fraction decreased cell viability in a dosedependent manner. Cancer is a major life-threatening disease among humans. Scorpion venoms are a natural source of molecules with anticancer activities. Several scorpion venom peptides have shown considerable anticancer effects against different types of cancer (Heinen and da Veiga, 2011; Ortiz et al., 2015). One important example is chlorotoxin, a peptide made up of 36 amino acids, first purified from the venom of the scorpion Leiurus quinquestriatus. It is considered to be a specific anticancer agent for the treatment of glioma. Researchers have also benetfied from the unique properties of this peptide for imaging. A bioconjugate of the chlorotoxin and a fluorescent compound is being used to determine the border of cancerous cells and helps clinicians in surgical operations (Veiseh et al., 2007) . Anticancer effects of scorpion venoms against leukemias are also being investigated. For example, the antiproliferative and apoptogenic activity of the venom of the Indian black scorpion (Heterometrus bengalensis) has been demonstrated against human leukemic cell lines U937 and K562 (Das Gupta et al., 2007). Studies regarding the anticancer activity of scorpion venoms have focused on buthid species because they are medically important, but, according to our results, venom of scorpions in the family Iuridae can also be considered as a potential source for anticancer peptides.

In conclusion, although Turkey possesses a rich scorpion fauna, scorpion venom-related studies are limited and concentrated on buthid species (Androctonus crassicauda, Buthacus macrocentrus, Mesobuthus gibbosus, Leiurus abdullahbayrami) (Caliskan et al., 2006; Ozkan et al., 2011; Caliskan et al., 2012; Diego-García et al., 2013; Erdeş et al., 2015) . In this paper, we present the first detailed biochemical characterization and bioactivity of P. kraepelini venom, which warrants further research that may result in the identification of new peptides with important pharmacological properties.

## Acknowledgments

The authors thank Kadir Boğaç Kunt for providing venom samples. LC-ESI-TOF measurements were carried out at the National Nanotechnology Research Center, Bilkent University. Microbial strains were kindly provided by Dr Arzu Çöleri Cihan (Ankara University, Turkey).

## Supplementary Material

Supplementary Table S1. Deconvoluted molecular weights of P. kraepelini venom peptides determined by LC-ESITOF- MS and MALDI-TOF-MS. Forty-eight distinct molecular masses were identified using the two methods in combination.
