# Supplementary material for: Peptidomic characterization and bioactivity of Protoiurus kraepelini (Scorpiones: Iuridae) venom
Source: Turk J Biol. 2018 Dec 10;42:490–7. doi: 10.3906/biy-1804-35 (PMC6451847; doi:10.3906/biy-1804-35)
Supplement: Supplementary Table S1. Deconvoluted molecular weights of P. kraepelini venom peptides determined by LC-ESITOF- MS and MALDI-TOF-MS. Forty-eight distinct molecular masses were identified using the two methods in combination. [file turkjbio-42-490-s001.pdf]

Supplementary Table S1. Deconvoluted molecular weights of *P. kraepelini* venom peptides determined by LC-ESI-TOF-MS and MALDI-TOF-MS. Forty-eight distinct molecular masses were identified using the two methods in combination.

MALDI-TOF MS	LC-ESI-TOF MS	
Deconvoluted mass (Da)	Deconvoluted mass (Da)	RT (min)
1470.8759	1059.2719	25.191
1584.8524	1147.0295	21.180
1628.8623	1252.3006	25.191
1776.6049	1471.8430	21.180
1869.9739	1512.8981	28.050
1886.2640	1629.9841	25.191
2030.3732	1657.9872	35.361
2073.3103	1990.9458	46.874
2089.4204	2072.9160	19.580
2128.2654	2129.9294	41.247
2165.5317	2157.3640	34.553
2380.5864	2382.1809	30.284
2409.3728	2458.2230	28.050
2425.7485	2536.2876	30.284
2507.7231	2580.3730	30.996
2557.0613	2604.3609	30.996
2579.9326	2648.2414	33.308
2692.0559	2722.8975	30.284
3224.3982	2903.3786	30.996
3405.8816	2979.8244	45.791
3802.0227	3033.5112	29.476
3990.5300	3316.9118	28.050
4050.1055	3383.6172	32.160
4191.1846	3408.9628	46.874
4623.2178	3479.8546	29.476
	3571.7598	34.553
	4393.2680	52.404
